# Composition of Essential Oils from Roots and Aerial Parts of *Carpesium cernuum* and Their Antibacterial and Cytotoxic Activities

**DOI:** 10.3390/molecules26071883

**Published:** 2021-03-26

**Authors:** Anna Wajs-Bonikowska, Janusz Malarz, Łukasz Szoka, Paweł Kwiatkowski, Anna Stojakowska

**Affiliations:** 1 Institute of General Food Chemistry, Faculty of Biotechnology and Food Sciences, Łódź University of Technology, Stefanowskiego Street 4/10, 90-924 Łódź, Poland; anna.wajs@p.lodz.pl; 2 Maj Institute of Pharmacology, Polish Academy of Sciences, Smętna Street 12, 31-343 Kraków, Poland; malarzj@if-pan.krakow.pl; 3 Department of Medicinal Chemistry, Faculty of Pharmacy with the Division of Laboratory Medicine, Medical University of Białystok, Mickiewicza Street 2D, 15-222 Białystok, Poland; lukasz.szoka@umb.edu.pl; 4 Department of Diagnostic Immunology, Chair of Microbiology, Immunology and Laboratory Medicine, Pomeranian Medical University in Szczecin, Powstańców Wielkopolskich Street 72, 70-111 Szczecin, Poland; pawel.kwiatkowski@pum.edu.pl

**Keywords:** alpha-pinene, *Carpesium cernuum*, 2,5-dimethoxy-*p*-cymene, Inuleae, monoterpenoids, thymohydroquinone dimethyl ether, thymol derivatives

## Abstract

*Carpesium cernuum* L., one of the two *Carpesium* species occurring in Europe, in the Far East and India, found use as a vegetable and a traditional medicinal remedy for several ailments. In the present study, compositions of essential oils distilled from roots and shoots of *C. cernuum* plants, cultivated in the open field, have been studied by GC-MS-FID supported by NMR spectroscopy. The analyses led to the identification of 120 compounds in total, of which 115 were found in aerial parts and 37 in roots of the plants. The major constituents found in the oil from shoots were: α-pinene (35%) and 2,5-dimethoxy-*p*-cymene (thymohydroquinone dimethyl ether, 12%), whereas 2,5-dimethoxy-*p*-cymene (55%), thymyl isobutyrate (9%) and thymol methyl ether (8%) predominated in the essential oil obtained from the roots. Antibacterial and cytotoxic activities of the essential oils distilled from *C. cernuum* were also tested. The essential oil from aerial parts of the plant demonstrated good inhibitory activity against *Staphylococcus aureus* ATCC 29213 and *Escherichia coli* ATCC 25922 (MIC: 15.6 μL/mL).

## 1. Introduction

Although plants of the genus *Carpesium* (Compositae, subtribe: Inuleae-Inulinae), have a long history of use as traditional herbal remedies and seasonal food in the Far East [1,2,3], they have no tradition of medicinal use in Europe. Majority of the species that belong to the genus are native to China, Japan and Korea, and some are endemic to China. *Carpesium cernuum* L. is a species native to Eurasia and is currently distributed in Europe, Asia and Australia [4,5,6,7]. The plant is a perennial herb, 20–80 cm tall, with an erect branched stem and solitary capitula (15–18 mm), without ray florets, which are subtended by many linear-lanceolate leaves. *C. cernuum* inhabits waste fields and mountain slopes below 3000 m [4,5]. The whole plant is medicinally utilized [1]. Recently, anti-inflammatory and a tumor migration inhibitory effects of extracts from *C. cernuum* have been described [8,9].

As a member of the Inuleae-Inulinae subtribe, the genus *Carpesium* is a close relative to such essential oil-bearing plants as *Inula helenium* L. and *Telekia speciosa* (Schreb.) Baumg. [10]. Although many studies on isolation of mono- and sesquiterpenoids from *C. cernuum* have been published before [1,11,12,13], data on composition of essential oils produced by the plant are not available. Essential oil from the herb of *Carpesium abrotanoides* L. was shown to induce apoptosis in hepatocellular carcinoma cells in vitro [14]. However, only 16 components of the examined oil were identified, and the established composition was significantly different from that described earlier [15]. A detailed analysis of essential oils from roots and aerial parts of *Carpesium divaricatum* Sieb. & Zucc. has been published recently [16], revealing the presence of numerous thymol derivatives, especially in the oil from roots of the plant.

The objective of the present study was to examine hitherto unknown chemical composition and biological activity of essential oils from roots and aerial parts of *C. cernuum* of European origin. Antibacterial activity of the essential oils against both Gram-positive and Gram-negative bacteria has been assessed, as well as cytotoxic activity towards normal and cancer cell lines.

## 2. Results

**Figure 1:**
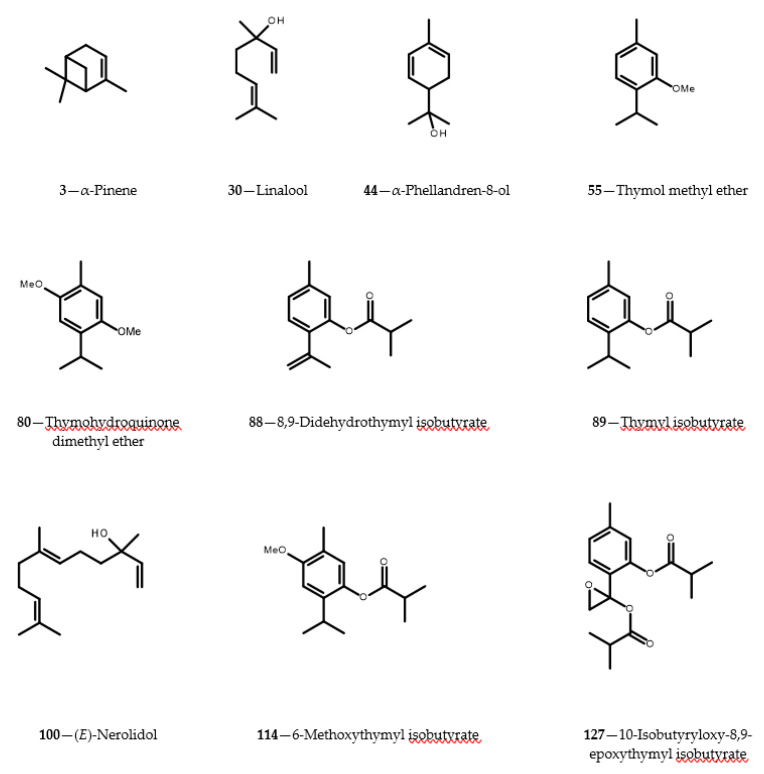
Structures of selected constituents (contents >3%) identified in essential oils from *Carpesium cernuum*.

Though yields of the essential oil produced by aerial parts of *C. cernuum* were low (0.015 ± 0.002%; see Table 1), 115 components of the oil, distilled from shoots of the plant collected at the beginning of flowering time, were identified by GC-MS. The identification was supported by NMR spectra analysis of the corresponding components isolated from the *C. cernuum* root essential oil. The identified compounds constituted 97.7% of the analyzed oil. Three compounds: α-pinene—the predominant constituent (35%, **3**), 2,5-dimethoxy-*p*-cymene (thymohydroquinone dimethyl ether, 12%, **80**) and linalool (4%, **30**) (see Figure 1) made up about 50% of the essential oil distilled from aerial parts of the plant. Eleven structurally diverse thymol derivatives (**55**, **80**, **88**, **89**, **114**, **115**, **120**, **121**, **126**, **127** and **129**) composed about 20% of the oil. Structures of six constituents of the essential oil remained unknown. The constituents could not be isolated and spectroscopically analyzed due to their minute amounts in the plant material.

Yield of the essential oil obtained from roots of *C. cernuum* was much higher than that from the aerial parts of the plant (0.170 ± 0.006%). In contrast to aerial parts, only 37 identified constituents made up nearly 99% of the root essential oil. The main compound found in the oil was 2,5-dimethoxy-*p*-cymene (**80**; c. 55%). Together with other thymol derivatives (**55**, **88**, **89**, **114**, **115**, **120**, **121**, **123**, **126**, **127** and **129**) it accounted for about 88% of the oil. Structure of one component of the root essential oil remained unresolved.

Antibacterial activities of the essential oils from aerial parts and from roots of *C. cernuum* were tested against the standard bacterial lines, both Gram-positive (*Staphylococcus aureus*, *Enterococcus faecalis*) and Gram-negative (*Escherichia coli*, *Klebsiella pneumoniae*, *Pseudomonas aeruginosa*, *Serratia marcescens*, *Acinetobacter baumanii*). Thymol, a monoterpenoid being found in numerous essential oils and having well-documented antimicrobial activity [17], was used as a reference compound. In general, the essential oil distilled from the aerial parts of *C. cernuum* demonstrated better bacteriostatic activity than that obtained from roots (see Table 2). *A. baumanii* ATCC 19606 and *S. aureus* ATCC 29213 were the most susceptible to both the examined essential oils and the thymol solution. Minimal inhibitory concentrations (MICs) against *S. aureus* were determined as 15.6 and 62.5 µL/mL for essential oils from *C. cernuum* aerial parts and roots, respectively. Growth of *S. marcescens* ATCC 13880 was inhibited only by the highest concentrations of the essential oils tested (MIC ≥ 250 µL/mL).

**Figure 2:**
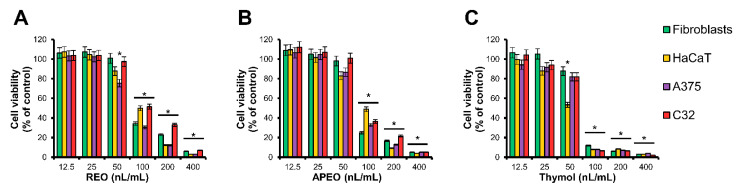
Cell viabilities of human skin fibroblasts, HaCaT keratinocytes and human melanoma cell lines A375 and C32, treated with different concentrations of *C. cernuum* essential oils and thymol for 48 h: (**A**) Essential oil from roots (REO), (**B**) essential oil from aerial parts (APEO) and (**C**) thymol. Data are presented as mean ± standard error of the mean (SEM) from three independent experiments. * *p* < 0.05 compared to control group.

To determine whether the examined essential oils are toxic to human skin, normal skin fibroblasts and keratinocytes were treated with increasing doses (12.5–400 nL/mL) of the *C. cernuum* essential oils for 48 h. In parallel, the experiments with human melanoma cells lines A375 and C32 were performed, to assess potential anti-cancer activity and selectivity of the cytotoxic effect. Cell viabilities were estimated using the 3-(4,5-dimethylthiazol-2-yl)-2,5-diphenyltetrazolium bromide (MTT) assay [18] (Figure 2), and the half-maximum inhibitory concentration (IC_50_) values of the essential oils and thymol towards the individual cell lines were calculated (Table 3). Our results indicated that toxicities of both root and aerial parts’ essential oils were almost equal. Moreover, toxic effects of the essential oils were slightly less pronounced than toxicity of thymol used as a reference agent. However, for both essential oils and thymol, no significant difference was found between toxic effect against normal and cancer cells.

## 3. Discussion

The tribe Inuleae of the Compositae (Asteraceae) comprises 62 genera of flowering plants divided into the two subtribes: Inuleae-Inulinae and Inuleae-Plucheinae [19]. *Blumea* spp., *Pulicaria* spp., *Dittrichia* spp. and *Inula* spp. have been the most frequently studied taxa of the Inuleae-Inulinae in respect of the production, composition and biological activity of the essential oils. The genera *Carpesium* and *Telekia*, though belonging to the same clade as *Inula*, are much less investigated. Only three papers on essential oils from *Carpesium* spp. have been published to date [14,15,16]. The two concerning *C. abrotanoides* [14,15] provided inconsistent data on the composition of the essential oils from the herb. This might be either due to different provenience of the plant material or due to different experimental procedures applied. In total, 16 and 44 components of the oil were identified with, respectively, eudesma-5,11(13)-dien-8,12-olide and β-bisabolene as major constituents. Neither thymol nor its derivatives were mentioned as components of the *C*. *abrotanoides* essential oils. As a result of biological activity testing, induction of apoptosis in human hepatocellular carcinoma cells (HepG2) by *C. abrotanoides* essential oil was experimentally proven [14].

The study on composition of essential oils obtained from roots and aerial parts of *C. divaricatum* cultivated in Poland [16] revealed marked differences in number and chemical structure of the compounds making up the two kinds of oil. Likewise, in *C. cernuum*, the main constituent of the essential oil from the aerial parts of *C. divaricatum* was *α*-pinene (40%, **3**). Thymol and its nine derivatives, including thymohydroqinone dimethyl ether (**80**, 2%), constituted nearly 5% of the oil. The content of thymol derivatives in the essential oil obtained from aerial parts of *C. cernuum* (APEO) was much higher (20%) than that found in *C. divaricatum*. Thymohydroquinone dimethyl ether was the most abundant thymol derivative present in the oil from aerial parts of the plant. Similar to *C. divaricatum*, roots of *C. cernuum* were a much better source of the essential oil than aerial parts. Likewise, the essential oils from the aerial parts, also the root essential oils of *C. cernuum* and root oil from *C. divaricatum,* differed in respect of the thymol derivatives content. Essential oil from roots of *C. cernuum* (REO) contained about 88% of the compounds with thymohydroquinone dimethyl ether as a main constituent (**80**, 55% of the oil), whereas thymol derivatives constituted about 61% of *C. divaricatum* root essential oil with 10-isobutyryloxy-8,9-epoxythymyl isobutyrate as a major component (**127**, 29%). *Telekia speciosa* (Schreb.) Baumg., the closest relative of *Carpesium* spp., produced essential oils rich in (*E,E*)-farnesol and (*E*)-nerolidol (leaves) or in isoalantolactone (flowers, roots). However, 10-isobutyryloxy-8,9-epoxythymyl isobutyrate (**127**) constituted 20% of the essential oil distilled from the flowers of the plant [20].

Essential oils with a high content of 2,5-dimethoxy-*p*-cymene are not rare within the Compositae. The compound was identified as a major component of essential oils distilled from plants representing different species of the Inuleae tribe (see Table 4).

2,5-Dimethoxy-*p*-cymene was also the main constituent of some essential oils from plants of the Eupatorieae tribe (Compositae) [31,32,33,34,35,36], especially, fresh leaves of *Ayapana triplinervis* (Vahl) R.M. King & H. Robinson (syn.: *Eupatorium luzoniense* Llanos, *E. triplinerve* Vahl) collected in Reunion Island, from which an essential oil was obtained containing 90% of the compound [35]. Recently, thymohydroquinone dimethyl ether from *A. triplinervis* has been proven to be active against Zika virus in non-cytotoxic doses [36]. Rhizomes and roots as well as achenes of a popular European medicinal plant *Arnica montana* L. (tribe Madieae of the Compositae) produced essential oils with a high content of thymohydroquinone dimethyl ether (20–60%), accompanied by smaller amounts of thymol methyl ether (4–27%), that induced apoptosis in cancer cells in vitro [37,38,39]. Roots of *Cyathocline purpurea* (D. Don) O. Ktze (Compositae), a rare Indian medicinal plant, contained essential oil with 2,5-dimethoxy-*p*-cymene as a major constituent (57%). The oil demonstrated bactericidal activity against Gram-positive bacteria [40]. Similar composition of essential oils could also be spotted in some species outside of the Compositae family, e.g., in *Cyclospermum leptophyllum* (Pers.) Sprague ex Britton and P. Wilson (Apiaceae) and *Aloe debrana* Christian (Xanthorrhoeaceae) [41,42].

The development of new antibiotics remains an ongoing challenge. Plant essential oils, and some individual compounds contained in them, may display good antimicrobial activity themselves or they can act synergistically with the antibiotics currently used in the therapy [43]. The essential oils distilled from dried roots and aerial parts of *C. cernuum* were active against both Gram-positive and Gram-negative bacteria, though their activity in comparison with that of thymol, used as a reference compound, was low. The determined MICs (see Table 2) were lower in the case of the essential oil obtained from the aerial parts of the plant. Thus, the simple positive correlation between thymol derivatives content or 2,5-dimethoxy-*p*-cymene content and the antibacterial activity of the oil should be excluded. It is worth to note that the activity of *C. cernuum* APEO against the standard line of *E. coli* is equal to that against *S. aureus,* a Gram-positive bacterium. *C. cernuum* REO was less active against *E. coli* which conforms to literature data on antibacterial activity of 2,5-dimethoxy-*p*-cymene-rich essential oils [29,40]. That activity is usually less pronounced against Gram-negative bacteria. The direct comparison of MICs determined for *C. cermuum* REO and APEO with the results obtained by the other research teams is difficult due to differences in the laboratory standards applied.

Antiseptic activity of compounds or essential oils should be associated with the lack of toxicity against human cells. Therefore, cytotoxicity of *C. cernuum* essential oils was tested in vitro using keratinocytes and fibroblasts derived from the human skin. Our data showed higher safety of the examined essential oils over thymol. However, the essential oils from *C. cernuum* were cytotoxic to the investigated cells in concentrations much lower (IC_50_: 0.072–0.107 µL/mL; Table 3) than those inhibiting bacterial growth (MIC: 12–250 µL/mL; Table 2). Finally, essential oils were tested in melanoma cell lines to assess anticancer activity. Unlike the essential oil from *C. abrotanoides* [14] and essential oils from *A. montana* [38,39], that demonstrated selective cytotoxicity against the cancer cells, *C. cernuum* REO and APEO did not show selective cytotoxicity towards the cancer cell lines used in this study. Cytotoxic activity of thymol was slightly higher than those of the tested *C. cernuum* oils. This result agrees with the previous study [44], indicating moderate toxicity of thymol towards a melanoma cell line. Selectivity of the cytotoxic effect in experiments using other types of cells could not be excluded and is worth further studies.

## 4. Materials and Methods

### 4.1. General Experimental Procedures

GC-MS-FID analyses of essential oils and their fractions were performed on a Trace GC Ultra Gas Chromatograph coupled with a DSQII mass spectrometer (Thermo Electron, Waltham, MA, USA). Simultaneous GC-FID and GC-MS analyses were performed using a MS-FID splitter (SGE Analytical Science, Ringwood, VIC, Australia). Mass range was 33–550 amu, ion source-heating: 200 °C, ionization energy: 70 eV. One microliter of essential oil solution (80% *v/v*) diluted in pentane:diethyl ether was injected in split mode at split ratios (50:1). Operating conditions: capillary column Rtx-1 MS (60 m × 0.25 mm, film thickness 0.25 μm), and temperature program: 50 °C (3 min)–300 °C (30 min) at 4 °C/min. Injector and detector temperatures were 280 and 300 °C, respectively. Helium was a carrier gas (constant pressure: 300 kPa). The relative composition of each essential oil sample was calculated from GC peak areas according to total peak normalization. ^1^H-NMR (700 MHz) and ^13^C-NMR (175 MHz) spectra for components of essential oils were recorded with a Bruker Avance II Plus 700 MHz spectrometer (Bruker Corp., Billerica, MA, USA) in CDCl_3_, with tetramethylsilane (TMS) as an internal standard.

### 4.2. Plant Material

Seeds of *Carpesium cernuum* L. were delivered by the Anastasie Fătu Botanical Garden of the Alexandru Ioan Cuza University in Iaşi (Romania). The seeds were collected from plants growing in the wild in the natural reserve Poiana cu Cetate (Pădurea Bârnova Natura 2000 site, Iaşi County) and were sown at the end of March 2017. Seedlings and young plantlets were grown in a glasshouse of the Garden of Medicinal Plants, Maj Institute of Pharmacology PAS in Krakow, under controlled conditions (temperatures by day 18–38 °C, by night 12–18 °C), without any chemical treatment. In the third week of May, the plants were transferred into the open field. Aerial parts and roots of the plants were collected at the second year of growth, in the beginning of the flowering period (July 2018), and dried under shade at room temperature. Voucher specimen (5/18) was deposited in the collection kept at the Garden of Medicinal Plants, Maj Institute of Pharmacology, Kraków, Poland. The dry plant material was stored no longer than five months before analysis.

### 4.3. Isolation of Essential Oil

Essential oils from the dried aerial parts (leaves, stalks, flowers; 4.3 kg) or roots (1.8 kg) of *C. cernuum* were obtained by hydrodistillation using a Clevenger-type apparatus. The process was conducted for 5 h using 100–550 g of plant material. The yellowish essential oils of specific strong odor were dried over anhydrous magnesium sulphate, and stored at 4 °C in the dark, until tested and analyzed.

### 4.4. Isolation and NMR Analysis of Major Volatile Components

The relatively high yield of the essential oil (0.17%) obtained from 1.8 kg of the dried roots allowed us to isolate its components that were difficult to identify by the GC-MS method. To isolate the volatiles of interest, the essential oil was flash-chromatographed (FC) on a glass column (500 × 30 mm) filled with silica gel 60 (0.040–0.063 mm, Merck, EM Science, NJ USA), starting the elution with *n*-hexane and gradually increasing the polarity by addition of diethyl ether. The elution was accelerated by means of pressurized nitrogen (flow rate 100 mL/min). The separation was monitored by TLC and GC-MS analysis. Twenty-two fractions (1–22) of the essential oil distilled from the roots of *C. cernuum* were obtained and analyzed by GC-MS-FID. Structures of 5 volatiles from the following fractions were confirmed using NMR spectroscopy (^1^H and/or ^13^C): fraction 6 (296 mg) thymohydroquinone dimethyl ether (93.3%), fraction 9 (170 mg) 6-methoxythymyl isobutyrate (93.8%), fraction 11 (93 mg) 6-methoxy-8,9-didehydrothymyl isobutyrate (64.2%), fraction 14 (388 mg) 7-isobutyryloxythymyl isobutyrate (85.7%) and fraction 18 (67 mg) 10-isobutyryloxy-8,9-epoxythymyl isobutyrate (69.7%).

### 4.5. Identification of Essential Oil Constituents

Volatiles from the essential oils were identified based on their MS spectra and their comparison with those from mass spectra libraries: NIST 2012, Wiley Registry of Mass Spectral Data 8th edition and MassFinder 4.1, along with the relative retention indices (RI) on DB-1 column (available from MassFinder 4.1) and on HP-5 column (available from NIST 2012). Isolated compounds were also identified by the comparison of their ^1^H-NMR and ^13^C-NMR spectral data with those of the compounds isolated previously in our laboratory or those from the literature.

### 4.6. Antibacterial Activity of Carpesium Cernuum Essential Oils

#### 4.6.1. Bacterial Lines and Culture Conditions

*Staphylococcus aureus* ATCC 29213, *Escherichia coli* ATCC 25922, *Enterococcus faecalis* ATCC 29212, *Klebsiella pneumoniae* ATCC 700603, *Pseudomonas aeruginosa* ATCC 27853, *Serratia marcescens* ATCC 13880 and *Acinetobacter baumanii* ATCC 19606 standard bacterial lines, from the collection of the Chair of Microbiology, Immunology and Laboratory Medicine, Pomeranian Medical University in Szczecin, were used to assess antimicrobial activities of the essential oils distilled from roots and aerial parts of *C. cernuum*. Prior to each experiment, the bacteria were seeded on Columbia agar medium with 5% sheep blood (bioMérieux, Warsaw, Poland) and incubated for 24 h at 37 °C in aerobic conditions. After that, the single bacterial colonies were transferred into the Tryptic Soy Broth (TSB; Merck, Darmstadt, Germany) and incubated for another 18 h at 37 °C in aerobic atmosphere.

#### 4.6.2. Determination of the Minimum Inhibitory Concentrations (MICs)

The MICs of *C. cernuum* essential oils against the selected Gram-positive and Gram-negative bacteria were determined using a broth microdilution method, according to the recommendations of the Clinical and Laboratory Standards Institute (protocol M07-A9) [45]. Bacterial suspensions in Mueller-Hinton broth (MHB; Merck, Darmstadt, Germany) at the final concentrations of 10^6^ CFU/mL were used in all experiments. Stock solutions of the essential oils (250.0 μL/mL) were prepared with 1% Tween 80 (*v/v*; Merck, Darmstadt, Germany) and a reference compound—thymol (Ernesto Ventos S.A., Barcelona, Spain)—was solubilized using 2% dimethyl sulfoxide (DMSO; *v/v*; Loba Chemie, Mumbai, India). Series of dilutions (from 1 to 250 μL/mL) were prepared by diluting the stock solutions with MHB. To each well in a 96-well microplate, containing 50 μL of the essential oil or thymol solution, 50 μL of the bacterial suspension was added. After 18 h of incubation at 37 °C, each well was spiked with 20 µL of 0.02% resazurin (Merck, Darmstadt, Germany) solution. The color change from blue to pink, after 3 h of incubation with resazurin, indicated the presence of viable bacteria. To exclude an inhibitory effect of Tween 80 and DMSO on the bacterial strain’s growth, control assays with MHB and MHB containing 1% (*v/v*) Tween 80 or 2% (*v/v*) DMSO were performed, as well as the MHB sterility control. All tests were run in duplicate.

### 4.7. Cytotoxic Activity of Essential Oils from Carpesium Cernuum

#### 4.7.1. Cell Lines and Culture Conditions

HaCaT keratinocytes were purchased from AddexBio (San Diego, CA, USA). Melanoma cells A375 were obtained from Sigma-Aldrich Co. (St. Louis, MO, USA). Melanoma cells C32 and normal human skin fibroblasts CCD25Sk were purchased from American Type Culture Collection (ATCC; Manassas, VA, USA). The cells were maintained in DMEM (PAN-Biotech GmbH, Aidenbach, Germany) supplemented with 10% fetal bovine serum (Gibco FBS; ThermoFisher Scientific, Waltham, MA, USA) and 1% penicillin/streptomycin (ThermoFisher Scientific, Waltham, MA, USA) at 37 °C in a 5% CO_2_ incubator.

#### 4.7.2. Cell-Viability Assay

Essential oils and thymol (Sigma-Aldrich Co, St. Louis, MO, USA) were solubilized with dimethyl sulfoxide (DMSO) and stored at −20 °C for up to one month. Final concentration of DMSO in culture medium never exceeded 0.1% and the same concentration of DMSO was used in control.

Viability of cells was determined by the MTT assay [18,46]. Cells were plated in 96-well plates at 1 × 10^4^ cells per well and allowed to adhere for 24 h. Afterwards, the cells were treated with the respective concentration of essential oils or thymol and incubated for 48 h. MTT (3-(4,5-dimethylthiazol-2-yl)-2,5-diphenyltetrazolium bromide) (Sigma-Aldrich Co., St. Louis, MO, USA) solution was added to each well and the cells were incubated at 37 °C for 4 h. Then, the medium was removed, and formazan crystals were dissolved in 100 μL of DMSO and 12.5 μL of Sorensen’s glycine buffer on a plate shaker. The absorbance was measured at 570 nm on a microplate reader and the results were expressed as a percentage of the absorbance measured in control cells.

Half-maximum inhibitory concentration (IC_50_) values were calculated by nonlinear regression analysis using GraphPad Prism version 7.04 (GraphPad Software, San Diego, CA, USA). The results were submitted to statistical analysis using one-way analysis of variance (ANOVA) followed by Tukey’s test, accepting * *p* < 0.05 as significant vs control.

## 5. Conclusions

This is the first study on composition and biological activity of the essential oils extracted from roots and aerial parts of *C. cernuum* of European origin. The examined oils differed in composition and antibacterial activity. The essential oil from the plant roots was rich in thymol derivatives, especially thymohydroquinone dimethyl ether (2,5-dimethoxy-*p*-cymene) of proven antiviral activity (55% of oil). The essential oil from aerial parts, of very complex composition, was rich in *α*-pinene and demonstrated better antibacterial activity than that determined for the root essential oil. The oils at the concentrations ≥100 nL/mL were cytotoxic in vitro to both cancer and normal cell lines used in the study.

## Figures and Tables

**Table 1:** Chemical composition of essential oils from aerial parts and roots of *Carpesium cernuum* L.

No	Compound	Aerial Arts	Roots	RI ^1^	RI ^2^
		Amount (%)			
1	n-Hexanal	0.2		775	769
2	(*E*)-2-Hexenal	1.7		826	822
**3**	**α-Pinene**	**34.7**	**0.1**	**932**	**932**
4	Camphene	0.1		940	943
5	Thuja-2,4(10)-diene	0.3		944	957
6	Octane-2,3-dione	tr		962	967
7	6-Methylhept-5-en-2-one	0.5		963	972
8	1-Octen-3-ol	0.9		964	972
9	β-Pinene	0.6		966	972
10	2-Pentylfuran	0.7		977	977
11	Myrcene	0.3		981	979
12	(*E*)-2-(2-Pentenyl)furan	0.2		985	983
13	m-Cymene	0.1		993	999
14	α-Terpinene	0.2		1007	1013
15	*p*-Cymene	0.2		1010	1015
16	1,1,3-Trimethylcyclohexan-3-one	0.2		1011	1019
17	4,6-Dimethylhept-5-en-2-one	0.3		1016	1119
18	Limonene	0.4		1019	1025
19	(*Z*)-β-Ocimene	0.1		1026	1029
20	2,6-Dimethylhept-5-enal	0.1		1034	1036
21	(*E*)-β-Ocimene	0.1		1038	1041
22	γ-Terpinene	0.3		1048	1051
23	*trans*-Linalooloxide (furanoid)	0.2		1057	1058
24	Non-1-en-3-ol	0.2		1068	1064
25	Camphen-6-ol	0.1		1072	1070
26	*cis*-Linalooloxide (furanoid)	tr		1075	1072
27	p-Cymenene	tr		1076	1076
28	Terpinolene	0.1		1078	1082
29	n-Nonanal	0.3		1084	1083
**30**	**Linalool**	**4.3**		**1088**	**1086**
31	Oct-1-en-3-yl acetate	tr		1096	1096
32	α-Cyclocitral	0.1		1097	1103
33	α-Campholenal	0.4		1104	1105
34	Perillene	0.2		1106	1109
35 + 36	Camphor + Unknown (MS: 123/86/119 M)	0.2		1120	1123
37	*trans*-Pinocarveol	0.4		1123	1126
38	*p*-Mentha-1,5-diene-8-ol	tr		1125	1127
39	*cis*-Verbenol	0.1		1129	1132
40	*trans*-Verbenol	0.2		1130	1137
41	*trans*-Pinocamphone	0.4		1138	1140
42 + 43	β-Phellandren-8-ol + 4-Ethylbenzaldehyde	0.4		1148	1143 + 1147
**44 + 45**	**α-Phellandren-8-ol + (2*E*)-4-(2-Methyl-1-cyclohexen-1-yl)-2-butenal**	**0.4**	**5.6**	**1149**	**1150 + 1149**
46	Terpinen-4-ol	0.5		1162	1164
47	Dimethylsiloxane pentamer (artifact)	0.1		1169	1169
48	α-Terpineol	1.0		1174	1176
49	Myrtenol	0.1		1181	1178
50	n-Decanal	2.2		1187	1180
51	Dihydrocarveol (Isomer 2)	tr		1193	1193
52	β-Cyclocitral	0.3		1197	1195
53	*trans*-Carveol	0.1		1202	1200
54	Nerol	0.1		1214	1210
**55**	**Thymol methyl ether**	**0.5**	**8.4**	**1216**	**1215**
56	β-*apo*-8-Carotenal	0.1		1236	1236
57	Geraniol	0.5		1238	1238
58	α-Ionene	0.1		1243	1255
59	Thymol	tr	tr	1269	1267
60	Carvacrol	0.1		1277	1278
61	Dihydroedulan I	0.2		1280	1290
62	Dihydroedulan II	0.4		1283	1296
63	Theaspirane (Isomer 1)	0.1		1290	1299
64 + 65	Myrtenyl acetate + Theaspirane (Isomer 2)	0.2		1305	1313 + 1313
66	γ-Pyronene	0.1		1316	1336
67	*cis*-Edulan	tr		1320	1328
68	Unknown (MS: 79/77/107 M178)	0.1		1324	
69	Dehydro-ar-ionene	0.1		1337	1336
70	α-Longipinene	0.1	tr	1350	1360
71	3-Hydroxy-2,4,4-trimethylpentyl 2-methylpropanoate	tr		1356	1381
72	(*E*)-β-Damascenone	0.2		1363	1361
73	α-Longicyclene		tr	1369	1382
74	Unknown (MS: 79/95/107/178 M204)	0.3		1370	–
75	1,2-Dihydro-1,4,6-trimethylnaphthalene	0.1		1372	1373
76	1,2-Dihydro-1,5,8-trimethylnaphthalene	0.1		1375	1376
77	1,4-Dimethoxy-2-tert-butylbenzene	tr		1382	1398
78	β-Ionol	0.1		1390	1400
79	7,8-Dihydro-β-ionone	0.2	0.2	1393	1413
**80**	**2,5-Dimethoxy-*p*-cymene** **(Thymohydroquinone dimethyl ether) ^3^**	**11.6**	**54.8**	**1405**	**1399**
81	2-tert-Butyl-1,4-dimethoxybenzene		0.3	1410	1400
81 + 82	2-tert-Butyl-1,4-dimethoxybenzene + Unknown: (MS: 105/119/77/147 M194)	0.4		1417	1400
83	Unknown (MS: 123/121/179 M194)	0.1		1421	–
84	Dihydropseudoionone	0.2		1433	1434
85	*trans*-α-Bergamotene	tr	0.3	1436	1434
86	epi-β-Santalene	0.4	tr	1443	1446
87	β-Santalene	1.0		1459	1458
**88**	**8,9-Didehydrothymyl isobutyrate**	**3.2**	**2.8**	**1464**	**1458**
**89**	**Thymyl isobutyrate**	**0.6**	**9.0**	**1465**	**1462**
90	(*E*)-β-Ionone	0.9		1472	1466
91	Neryl isobutyrate	0.1	1.3	1477	1468
92	α-Terpinyl isobutyrate		0.1	1477	1498
93	(*3Z,6E*)-α-Farnesene	0.5		1483	1480
94 + 95	α-Selinene + Unknown (MS: 99/121/155 M204)	0.9		1492	1494
96	(*3E,6E*)-α-Farnesene	0.4		1496	1498
97	β-Bisabolene	0.3	tr	1501	1503
98	*cis/trans*-Calamenene	0.1		1521	1517
99	(*E*)-β-Caryophyllene oxide		tr	1535	1545
**100**	**(*E*)-Nerolidol**	**3.3**	**0.2**	**1552**	**1553**
101	Neryl isovalerate	0.6	0.6	1559	1565
102	Geranyl isovalerate	0.2	0.1	1565	1573
103	Caryophyllene epoxide	0.2	0.1	1571	1578
104	Isoaromadendrene epoxide	0.1	tr	1574	1592
105	Widdrol	0.1		1596	1601
106	Acora-2,4(15)-dien-11-ol	0.1		1613	1616
107	α-Acorenol	0.1	0.2	1621	1623
108	1-*epi*-Cubenol	tr	tr	1624	1623
109	β-Acorenol		0.8	1632	1626
110	β-Eudesmol	0.4	tr	1632	1643
111	Tetradecan-13-olide	0.2		1638	1643
112	5β,7βH,10α-Eudesm-11-en-1α-ol	0.3	0.2	1642	–
113	*cis*-Eudesma-4,11-dien-8-ol	tr	0.1	1656	1648
**114**	**6-Methoxythymyl isobutyrate ^3^**	**3.3**	**5.2**	**1662**	**1658**
115	6-Methoxy-8,9-didehydrothymyl isobutyrate ^3^	0.1	0.1	1676	1676
116	(*E,E*)-Farnesol	0.3		1703	1694
117	Benzyl Benzoate	tr		1731	1730
118	Isobutyl phthalate (artifact)	5.4	0.3	1834	1840
119	Benzyl salicylate	0.1		1842	1847
120	9-Isobutyryloxythymyl isobutyrate	0.8	1.0	1882	1891
121	10-isobutyryloxy-8,9-dehydrothymyl isobutyrate	1.1	1.3	1886	1891
122	Eudesma-5,11(13)-dien-8,12-olide	0.1	0.4	1906	1891
123	7-Isobutyryloxythymyl isobutyrate ^3^		0.1	1921	1930
124	Butyl phthalate (artifact)	0.5		1921	1909
125	Hexadecanoid acid	0.6		1955	1942
126	9-(2-Methylbutyryloxy)thymyl isobutyrate	0.1	0.1	1968	1970
**127**	**10-Isobutyryloxy-8,9-epoxythymyl isobutyrate ^3^**	**0.5**	**5.1**	**1991**	**2036**
128	Unknown (MS: 177/150/71 M290)		0.4	2049	–
129	10-(2-methylbutyryloxy)-8,9-epoxythymyl isobutyrate	0.1	0.4	2077	2056
130	*trans*-Phytol	0.4		2101	2104
	**Sum of identified**	**97.7**	**99.6**		
	**Yield of the essential oil (%)**	**0.015 ^4^**	**0.170 ^5^**		

^1^ Experimental retention index (RI). ^2^ Literature retention index calculated on DB-1 column. ^3^ Compound identified by RI, MS, ^1^H-NMR and ^13^C-NMR. ^4^ Mean value for two 5 h distillations. ^5^ Mean value for three 5 h distillations; tr < 0.05% (trace).

**Table 2:** Antibacterial activities of essential oils from roots (REO) and aerial parts (APEO) of *Carpesium cernuum* L.

Bacterial Cell Line	*C. Cernuum* REO	*C. Cernuum* APEO	Thymol
	Minimal Inhibitory Concentration (MIC)		
	(µL/mL)		(µg/mL) ^1^
*Staphylococcus aureus* ATCC 29213	62.5 ± 0.0	15.6 ± 0.0	0.9 ± 0.0
*Escherichia coli* ATCC 25922	125.0 ± 0.0	15.6 ± 0.0	7.5 ± 0.0
*Enterococcus faecalis* ATCC 29212	125.0 ± 0.0	93.8 ± 44.2	1.9 ± 0.0
*Klebsiella pneumoniae* ATCC 700603	125.0 ± 0.00	62.5 ± 0.0	15.0 ± 0.0
*Pseudomonas aeruginosa* ATCC 27853	250.0 ± 0.0	93.8 ± 44.2	7.5 ± 0.0
*Serratia marcescens* ATCC 13880	>250	250.0 ± 0.0	30.0 ± 0.0
*Acinetobacter baumanii* ATCC 19606	11.7 ± 5.5	11.7 ± 5.5	1.9 ± 0.0

^1^ 1 μg/mL of thymol corresponds to c. 0.001 μL/mL (weighted at the melting point of thymol).

**Table 3:** The half-maximum inhibitory concentration (IC_50_) values (nL/mL) calculated for the essential oils from roots (REO) and aerial parts (APEO) of *Carpesium cernuum* L. and thymol towards the human skin fibroblasts, keratinocytes (HaCaT) and melanoma cell lines (A375, C32).

Cell Line	IC_50_ (nL/mL)		
	*C. Cernuum* REO	*C. Cernuum* APEO	Thymol ^1^
Fibroblasts	83.02 ± 6.51	75.65 ± 4.83	65.62 ± 5.24
HaCaT	90.26 ± 6.62	82.14 ± 5.02	49.85 ± 4.12
A375	71.66 ± 5.12	75.9 ± 5.78	62.55 ± 4.62
C32	107.2 ± 6.40	82.32 ± 7.11	60.39 ± 6.45

^1^ 1 nL/mL of thymol corresponds to 1 μg/mL.

**Table 4:** Contents of 2,5-dimethoxy-*p*-cymene in essential oils (EOs) from selected species of the Inuleae.

Family/Tribe-Subtribe	Species	Plant Organ	2,5-Dimethoxy-*p*-cymene Content in EO	Literature
Compositae/Inuleae-Inulinae	*Blumea perrottetiana* DC.	Aerial parts	30%	[21]
	*Blumea virens* DC.	Roots	28%	[22]
	*Pulicaria mauritanica* Coss.	Roots	37%	[23]
Compositae/Inuleae-Plucheinae	*Laggera alata* (D. Don) Sch. Bip. ex Oliv.	Herb	44%	[24]
	*Laggera crispata* (Vahl) Hepper &Wood	Different organs	22–75%	[25,26]
	*Laggera pterodonta* (DC.) Sch. Bip ex Oliv.	Aerial parts	31–79%	[27,28]
	*Laggera tomentosa* Sch. Bip. ex Oliv. et Hiern	Stem bark and roots	57–65%	[29]
	*Sphaeranthus indicus* Kurz.	Roots and herb	27–28%	[30]

## Data Availability

The data presented in this study are available on request from the authors.

## Sample Availability

Samples of the compounds are not available.
